# Targeted delivery of nanomedicines for promoting vascular regeneration in ischemic diseases

**DOI:** 10.7150/thno.73421

**Published:** 2022-08-29

**Authors:** Jie Zhuang, Xiangyun Zhang, Qiqi Liu, Mingsheng Zhu, Xinglu Huang

**Affiliations:** 1 School of Medicine, Nankai University, Tianjin 300071, China.; 2 Key Laboratory of Bioactive Materials for the Ministry of Education, College of Life Sciences, State Key Laboratory of Medicinal Chemical Biology, and Frontiers Science Center for Cell Responses, Nankai University, Tianjin 300071, China.; 3 Joint Laboratory of Nanozymes, College of Life Sciences, Nankai University, Tianjin 300071, China.

**Keywords:** nanomedicines, targeted delivery, vascular regeneration, theranostics, ischemic diseases

## Abstract

Ischemic diseases, the leading cause of disability and death, are caused by the restriction or blockage of blood flow in specific tissues, including ischemic cardiac, ischemic cerebrovascular and ischemic peripheral vascular diseases. The regeneration of functional vasculature network in ischemic tissues is essential for treatment of ischemic diseases. Direct delivery of pro-angiogenesis factors, such as VEGF, has demonstrated the effectiveness in ischemic disease therapy but suffering from several obstacles, such as low delivery efficacy in disease sites and uncontrolled modulation. In this review, we summarize the molecular mechanisms of inducing vascular regeneration, providing the guidance for designing the desired nanomedicines. We also introduce the delivery of various nanomedicines to ischemic tissues by passive or active targeting manner. To achieve the efficient delivery of nanomedicines in various ischemic diseases, we highlight targeted delivery of nanomedicines and controllable modulation of disease microenvironment using nanomedicines.

## Introduction

Blood vessels mediating the balance of nutrients, oxygen and waste products, are one of the most important organ systems throughout the body 1, 2. Vascular dysfunction can initiate a serial of clinical diseases 3, 4. Ischemic diseases are a group of diseases initiated by vascular dysfunction, including ischemic heart disease, ischemic cerebral disease and peripheral ischemic vascular disease, which are a leading cause of global morbidity and mortality 5-8. Treatment of ischemic diseases remains challenging in clinics. The essential factor to address the challenges is to re-perfuse ischemic tissues by the regeneration of functional vasculature network 9-11. In fact, the intrinsic driving force of new vessel formation is present when suffering from stimulus of specific signals in the body. Hypoxia, a physiological phenomenon to master oxygen adaptive changes, occurs in tissues accompanying with ischemic status 12-14. In response to hypoxic episodes, the cells secret multiple pro-angiogenesis factors to induce vascular regeneration, such as vascular endothelial growth factor (VEGF) 15, fibroblast growth factor (FGF), platelet-derived growth factor (PDGF) and angiopoietins (Angs, including Ang-1, Ang-2 and Ang-4) 16. However, this spontaneous vascular formation is commonly limited to several tenths of micrometers per day. In other words, the physiological response of vascular regeneration is slow and tends to take days to weeks 17. In addition, a limited capacity to appropriately response to hypoxic stress paradoxically appears while the ischemic tissues are chronically/constantly exposed to hypoxia. Thus, the spontaneous physiological response is largely insufficient to meet the requirement of re-vascularization of ischemic tissues.

Various strategies have been developed to induce new vessel formation. Delivery of cytokines, such as VEGF, has been considered as an efficient strategy to induce angiogenesis 18. However, blood vessel formation is a highly organized process *in vivo* 15, 19, while this exogenous stimulus tends to overload the modulation, resulting in extra proinflammatory diseases and promoting tumor growth. The major reason is that the formation of new blood vessels is a sequential process, mediated by not only one specific factor but the synergistic actions of various angiogenesis factors. For example, during angiogenesis, VEGF is needed early to initiate new vessel formation 20, 21 while PDGF is required later to stabilize the neovessels 22. As such, the factors must be in the right place at the right time to orchestrate the actions. Rapid progress in nanoparticle synthesis has provided a broad range of nanoscale agents 23-26. Specifically, nanoparticles can serve as drug carriers for loading multiple factors simultaneously. Sequential delivery of angiogenic factors to improve revascularization in ischemic diseases has been revealed. Recently, we introduced a particle-based stem cell technology by integration of pro-angiogenic “cocktail” factors secreted from hypoxic mesenchymal stem cells (MSCs) into particles with cell membrane-derived surface coating 27. The controlled release of various pro-angiogenic factors provided superior revascularization effects on ischemic tissues in different ischemic models. Additionally, tissue ischemia results in enhanced permeability 28 and leakage of vascular endothelial cells and induced surface markers overexpression 29, providing a design principle of nanomedicines for targeting ischemic tissues. Thus, this review will summarize the nanomedicines that have been used for the restoration of vascular structure and novel approaches that are applicable to harness the angiogenic potential of nanomedicines in different contexts.

## Clinical symptoms and animal models

### Myocardial infarction

Myocardial infarction (MI) is mainly caused by the stenosis and occlusion of the coronary artery, which leads to inadequate blood supply to the cardiac tissues and subsequently myocardial hypoxia. It was estimated that coronary atherosclerosis caused ~12 million deaths annually due to either ST-segment elevation MI (STEMI, complete blockage of a major coronary artery) or non-ST-segment elevation MI (NSTEMI, partial obstruction of a major or minor coronary artery) 30. Cardiac ischemia, if not treated promptly, elicits a continuum of irreversible pathophysiological responses, including cell apoptosis, regional contractile dysfunction, myocardium fibrosis and substantial portion of myocardium loss 31-33. The repair of injured myocardium is almost impossible by physiological regeneration of cardiac muscle cells as the innate proliferative capacity of cardiomyocytes is very low 34, 35. Prolonged myocardial ischemia leads to loss of endothelial cell functions, microvascular dysfunction, and finally adverse cardiac function recovery. Thus, improving new vascular formation to meet oxygen and nutrient supply of infarcted tissues is regarded as a key strategy for ischemic repair.

The most common used myocardial ischemia model in laboratory is the permanent ligation of the left anterior descending artery (LAD) 36, 37. Once closing the LAD, no further blood flow is allowed in this area and an ischemia region is formed which can be seen immediately. The surgical procedure initiated the pathobiological and pathophysiological process occurring in infarction-related myocardial ischemia. The model is convenient for investigating the acute and chronic pathophysiological processes in myocardial ischemia 38. As another ischemia-relevant model, cardiac ischemia reperfusion model was initially applied experimentally *ex vivo* and then in dogs in 1988 39. Similar to permanent ligation, a left-sided thoracotomy is performed and the suture can be cut resulting in reperfusion after a period of ischemia. The ischemia time varies from 15 minutes to 2 hours, with 30 minutes to be the most commonly used 38, 40. With reperfusion, oxygen become rapidly increased, allowing a burst of free radical production for tissue damage 41. Both permanent ligation and ischemia reperfusion are valuable tools for the study of MI. Compared to permanent ligation, ischemia reperfusion may be closer with clinical cases since timely reperfusion of myocardial ischemia is the front-line treatment choice. Recently, cryoablation method by a precooled probe applied to the epicardial surface is widely used in mice to generate precisely controlled size, shape and location of infarcts 42. This approach is suitable for the study of infarct mechanics and functional regeneration strategies. Because of the relative low animal-to-animal variability, this model provides high reproducibility of infarcts and less invasive 43.

### Ischemic stroke

Ischemic stroke is caused by transient or permanent occlusion of a cerebral blood vessel eventually leading to brain infarction 44. The infract size of brain and neurological outcome after focal cerebral ischemia depend on the duration and severity of ischemia and the localization of the infract 45, 46. Following ischemic stroke, regions of the brain tissues suffering from ischemia, elicit a cascade of changes and vascular remolding. The traditional therapy for ischemic stroke mainly comprises the use of antithrombotic drugs and neuroprotectant to improve the blood circulation and protect neurons, respectively 47. Clinically, antithrombotic agents can be generally divided into antiplatelet and anticoagulant drugs, which are used to prevent thrombosis or to dissolve the existing thrombus 48. Neuroprotective agents could exert protective effects on neuron at different stages of stroke in clinical trials 49. Although some of mentioned drugs have used clinically, there are still some problems needed to be resolved. For example, the low thrombus targeting efficiency would lead to the increased risk of bleeding and intracerebral hemorrhage.

Obviously, ischemic stroke is highly heterogeneous and complicated, which leads to experimental models of stroke can only represent specific aspects of this complex disease 50. Since thromboembolism is the main cause of stroke in humans, thromboembolic stroke models closely stand for the clinical setting of stroke. Embolic ischemia inducing by injecting autologous thrombi into desired arteries has the potential to screen thrombolytic drugs. However, significant variability in the infarct volume, heterogenous nature of lesion development and increased rate of mortality and cerebral hemorrhage limit the use of the thromboembolic model 51. The most commonly used *in vivo* experiment model in stroke research is the intraluminal suture middle cerebral artery occlusion (MCAO) model, which does not need craniectomy 52. A monofilament is introduced into the internal carotid artery to induce vessel occlusion 53. This method was built and first reported by Koizumi and colleagues in rats and the model has also been adapted for mice later on. Generally, the suture model can be used to create permanent ischemia or ischemia-reperfusion by withdrawal of the filament. Technically, the MCAO model is less invasive and does not require craniectomy and thus avoids damage to cranial structures. In addition, hypertension is the most important modifiable risk factor for human stroke and therefore the use of hypertensive animals for stroke research is the most relevant choice to mimic an accurate situation of clinical reality 54, 55. The stroke-prone spontaneously hypertensive rat model is the most commonly used and it is currently by far the best animal model for lacunar stroke in humans due to the similarity with human stroke 56. Besides, other models such as the photothrombosis model are used widely due to its minimal surgical intervention, less invasiveness, high reproducibility of the lesions, and low mortality of the animals. This model was built by a beam of light with a specific wavelength directed into artery after intravenous injection of a photosensitizing dye, which triggered the generation of thrombosis and the breakdown of blood-brain barrier (BBB) within a few minutes 57.

### Hindlimb ischemia

Hindlimb ischemia is commonly caused by narrowing or blockage of arteries supplying blood to the lower limbs 58, 59. Since the prevalence of limb ischemia nearly equals to that of coronary artery disease, limb ischemia has become an important public health problem 60, 61. The clinical manifestation of the most advanced stage of lower limb ischemia showed rest pain and skin ulceration/gangrene, resulting in limb amputation 62, 63. The incidence of limb ischemia increases with age, which results in a significant economic burden and severely affects the quality of life of patients 64-66. In general, the lack of blood flow of tissues gradually deprives the oxygen and nutrients, leading to the impairment of muscle cell metabolism 67. Limb ischemia, if not effectively intervened, may lead to amputation. There is no better alternative to relieve ischemic symptoms and prevent limb loss at this stage of disease other than prompting revascularization. Current treatments including surgical bypass approach and angioplasty are regarded as the effective treatment for limb salvage 68, 69. But only about 30% of patients are eligible for limb-preserving surgical revascularization procedures 59. Besides, the traditional pharmacological treatments are not sufficient to alleviate the progression of disease.

The most frequently used hindlimb ischemia model is performed in murine, wherein surgical ligation of the femoral artery at a specific site triggers arteriogenesis in femoral collaterals proximally and angiogenesis in distal ischemic muscle 70-72. It can not only mimic aspects of human occlusive limb artery disease to investigate mechanisms of arterial pathophysiology and vascular regeneration, but also use to evaluate various therapeutic approaches in a reproducible manner 73, 74. Moreover, the hindlimb ischemia model can further be used to evaluate the vascular response of arteriogenesis and angiogenesis qualitatively and quantitatively. Besides, a double electrocoagulation can be performed in the femoral artery leading to severe ischemia and increased degree of tissue damage which make this model suitable to evaluate the therapeutic approaches for promoting both arteriogenesis and angiogenesis 75.

## Pathophysiological response to ischemia

### Tissue response to ischemia

**Figure 1:**
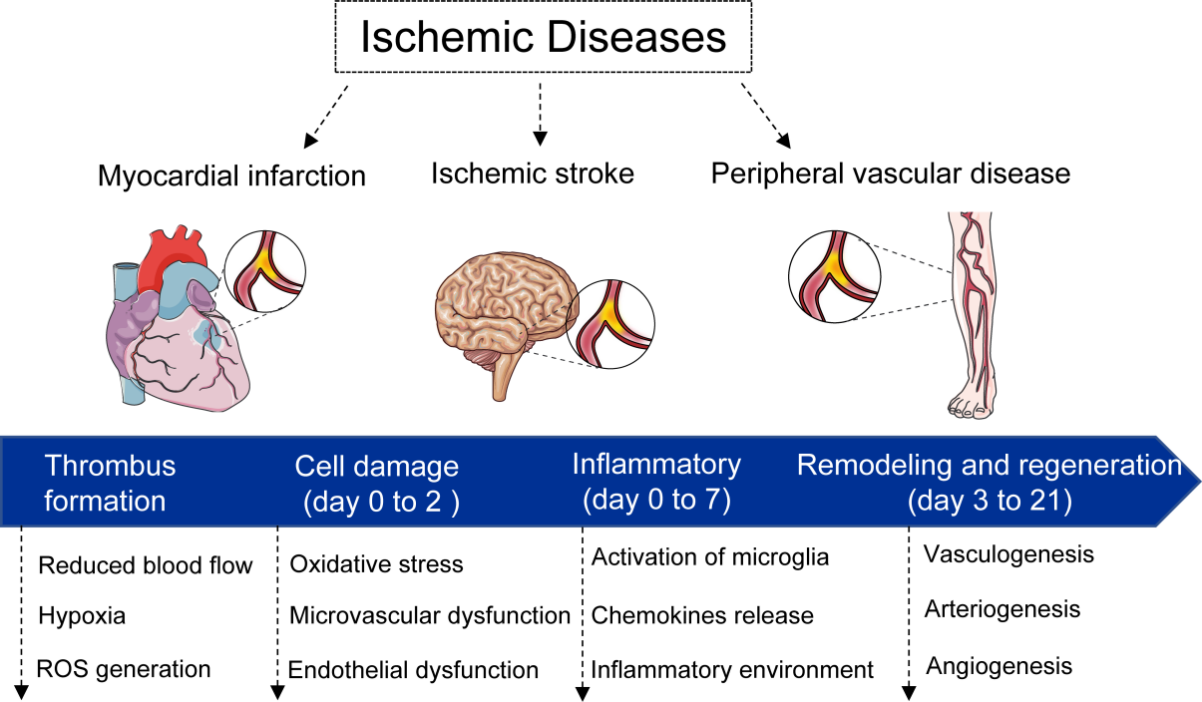
** Schematic diagram of ischemia diseases and physiological response.** In general, the responses can be divided into four phases: i) thrombus formation phase. Under some physiological and pathological factors, the vessels are occluded as a result of the thrombus formation; ii) cell damage phase (day 0 to 2). After 0-2 days, the accompanying of ischemic events such as hypoxia, hypoperfusion, metabolic dysregulation induce cell apoptosis and necrosis; iii) inflammatory phase (day 0 to 7). The inflammatory features appear. The migration and differentiation of myofibroblasts, and extracellular matrix proteins deposition, eventually lead to formation of a scar tissue to replace necrotic cells; iv) remodeling and regeneration phase (day 3 to 21). During this latter phase, new vessel formation and/or remodeling of the preexisting vasculature take place in ischemic zone.

Once occurrence of vascular occlusion, the surrounding tissues undergo a continuum of molecular, cellular, and extracellular responses. In general, the responses can be divided into four phases (**Figure 1**): i) thrombus formation phase. Under some physiological and pathological factors, the vessels are occluded as a result of the thrombus formation, which in turn results in the deficient supply of the oxygen and nutrients; ii) cell damage phase (day 0 to 2). After 0-2 days, the accompanying of ischemic events such as hypoxia, hypoperfusion, metabolic dysregulation induce cell apoptosis and necrosis; iii) inflammatory phase (day 0 to 7). The inflammatory features appear. Following the secretion of cytokines, chemokines and growth factors from damaged and inflammatory cells, the neutrophil, monocyte, and lymphocyte home and migrate into the necrotic tissues for the removal of dead cells. In addition, the inflammatory events induce metalloproteinases (MMPs) expression for the degradation of extracellular matrix (ECM) 76, 77. The migration and differentiation of myofibroblasts, and ECM proteins deposition, eventually lead to formation of a scar tissue to replace necrotic cells 78; iv) remodeling and regeneration phase (day 3 to 21). During this latter phase, new vessel formation and/or remodeling of the preexisting vasculature take place in ischemic zone.

### Mechanisms of vascularization and vascular remodeling

Understanding of the molecular mechanisms of blood vessel formation* in vivo* is a prerequisite for engineering vascular constructs or inducing vascularization from preexisting blood vessels. Vasculogenesis, arteriogenesis and angiogenesis are the main processes that are responsible for the formation of natural vascular networks 13, 32. Vasculogenesis is mainly involved in early embryogenesis 79. In the adult vascular system, angiogenesis and arteriogenesis play critical roles in both health and disease conditions, that are also the strategies for reconstructing vascularization in tissue engineering 15. Angiogenesis is responsive to hypoxia, whereas arteriogenesis is likely triggered by mechanical forces. Among the three vascular formation processes, angiogenesis is undoubtedly well-known due to its importance in tumor occurrence and development 80. Importantly, it is currently the major route to control vascularization in ischemic disease.

Angiogenesis that is defined as the formation of new vessels from pre-existing vascular bed, is the major process involved in vascular organization and remodeling. Angiogenesis is a highly controlled via an intricate balance of both pro-angiogenic and anti-angiogenic factors, but the process is a sequential, multistep process 13, 15, 19, 80. First, when stimulation of quiescent vessels with cytokines and chemokines released by hypoxic, inflammatory or tumor cells, the degradation of the basement membrane via extracellular proteinases such as MMPs, results in pericyte detachment and loosening of endothelial cell junction 81. Subsequently, VEGF increases the permeability of endothelial cell layer, permitting extravasation of plasma proteins to deposit a provisional ECM scaffold. This was followed by the formation of capillary buds and sprouts, terms as tip-cells, and migration of endothelial cells. FGF is one of the factors participating in the initiating phases of angiogenesis by promoting endothelial cells proliferation and migration 15, 82. Stalk cells behind the tip cells proliferate, elongate and form a lumen, and sprouts fuse with an adjacent vessel spout to establish a perfused neovessel. After the activation and proliferation stages, a nascent blood vessel must mature to become functional. Vessel maturation is mediated through the actions of growth factors such as Ang-1 or PDGF. On the basis of vessel types, differentiated smooth muscle cells or pericytes stabilize vessel structures and suppress endothelial cell growth. Smooth muscle cells are used to support arteries and veins, while pericytes enable capillary maturation.

### Hypoxia-mediated angiogenesis

Ischemia-induced angiogenesis is a physiological response to tissue hypoxia. At O_2_ concentrations < 6%, most tissues can trigger this response by stabilizing HIF-1α. HIF-1α involved in all steps of the post-ischemic revascularization process by controlling the expression of numerous major players involved in angiogenesis, including VEGF and its receptor VEGFR, C-X-C motif chemokine ligand 12 (CXCL12), Ang-2, PDGF, and stem cell factor (SCF) 83. However, the physiological adaptation does not always response to ischemia-hypoxic stress according to previous studies. For example, in some amputated limbs of patients, only the upregulated expression of HIF-1α is observed in acute ischemic tissues, whereas that of chronically ischemic tissues is decreased. In addition, hypoxia-responsive genes are adaptively upregulated under acute ischemic conditions, gradually undergo downregulation in chronic ischemia. In other words, the transition from acute to chronic hypoxia always happens along with ischemic process. The potential mechanism is complicated by the fact that the spatial and temporal distribution patterns of endogenously produced angiogenic responses in ischemic tissues, but a blunting of physiological adaptation response of cells due to prolonged/repeated hypoxic exposures might be involved. Spatio-temporally controlling the habituated response of cells could therefore provide a solution to overcome the limited ability of ischemic tissues, to effectively upregulate angiogenic signaling, in spite of prolonged exposure to hypoxia.

The advantages of mastering hypoxia for angiogenesis have been demonstrated by accumulated evidences. Pajusola *et al*. showed that adeno-associated virus (AAV) encoded HIF-1α obviously induced capillary sprouting by injection into skeletal muscle, whereas AAV-VEGF only induced endothelial proliferation without proper vessel formation 84. Furthermore, unlike VEGF, HIF-1α overexpression did not increase vascular leakiness in the treated muscle. These results indicated that stabilization of HIF-1α was superior to VEGF for angiogenesis in skeletal muscle via AAV gene transfection. Elson *et al*. also found that despite marked induction of hypervascularity, transgenic mice overexpressing HIF-1α did not induce edema, inflammation, or vascular leakage, which commonly occurred in VEGF-overexpressed transgenic mice 85. Agreement with these results, Trentin *et al*. also displayed that, in addition to superior angiogenic effect, HIF-1α activation can avoid common problems relevant with overexpression of individual angiogenic growth factors, such as formation of unstable/leaky vasculature.

Throughout the previous reports, delivery of isolated angiogenic growth factors (*e.g.*, VEGF) that directly stimulate vessel formation or maturation is no doubt major strategies for ischemic therapy. Although the approach has showed the positive outcomes in various studies, its disadvantages are also apparent 86, 87. Compared to direct delivery method, the hypoxia-mastered approach to indirectly regulate angiogenic growth factors has several advantages. Firstly, both vessel formation and maturation are regulated by a panel of angiogenic factors. Unlike the direct delivery of a specific angiogenic factor, hypoxia-inducible factors drive transcription activation of hundreds of genes involved in vascular process 83. The regulation of hypoxic status steers the whole revascularization process in ischemic tissues. Secondly, the production of angiogenic factors by hypoxic cells is well-controlled to guarantee that the concentration of angiogenic factors is in the physiological range, which is adapted to the requirements of different stages of vessel formation. Last but not the least, the secretion of angiogenic factors leads to the gradient microenvironment of growth factors, which is important for capillary morphogenesis.

## Treatment of ischemic diseases using nanomedicines

### Why nanomedicines?

While critical ischemic diseases happen, ischemia can lead to microvascular dysfunction, characterized by increased vascular permeability, endothelial cell inflammation, an imbalance between vasodilating and vasoconstricting factors and activation of coagulation and the complement system. During the ischemic period, vascular hypoxia leads to enhanced permeability and leakage of vascular endothelial cells through the lower of adenylate cyclase activity and intracellular cAMP levels, a physiological adaptive response to compensate the supply of oxygen and nutrients 88. Quantitatively, the vessel permeability is meaused by hydraulic conductivity, which is defined as how quickly a liquid will flow through the blood vessels. Experimental study found that the hydraulic conductivity of blood vessels after ischemia is 3.15 ± 0.10 cm· s^-1^ ·cm H_2_O^-1^ × 10^-7^, which is approximately 3-fold higher than sham control 89. Based on the elevated permeability of blood vessels during ischemia, the nanoparticles can gradually accumulate into the ischemic tissues over time, showing an enhanced permeability and retention (EPR) effect for nanoparticle mediated drug delivery 90. From this point, the targeting mechanism is similar with tumor passive targeting.

Moreover, it is critical to spatio-temporally guide an angiogenic response, i.e. control when and where it is induced, for how long and in what direction. Nanoparticle mediated drug delivery provides: i) a long-lasting drug release through the long-term retention time of nanoparticles; ii) a controlled release manner to avoid unwanted side effects, such as ectopic angiogenesis, vascular leakage, tumor formation, *etc.*; iii) controllable directionality of angiogenesis by localized delivery of angiogenic signaling at a target site (*e.g.* ischemic area, implant site). Whereas nanoparticles enable fine-tuned release of growth factors over time, this strategy is largely dependent on particle properties, which is not adequately suitable for the complex microenvironment in sequential process of ischemic disease. Instead, ischemic microenvironment-responsive drug delivery may represent a more advanced way to achieve personalized therapy.

### Targeting ischemia using nanomedicines

**Figure 2:**
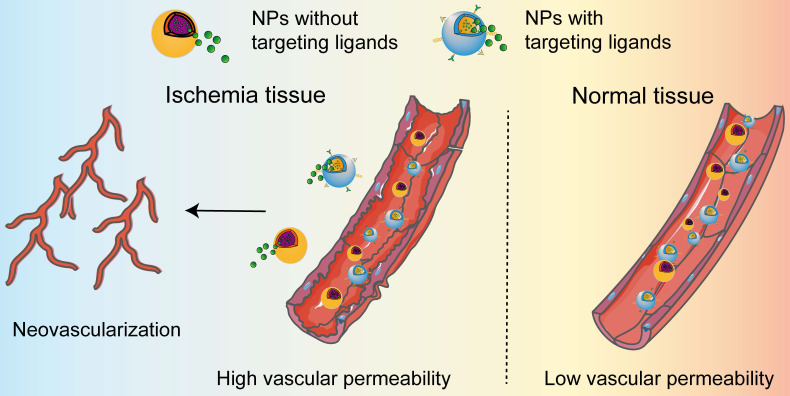
** Delivery of nanomedicines in ischemic tissues.** The permeability of nanoparticles is low in normal vessels. When ischemic diseases occur, ischemia leads to vascular dysfunction, one manifestation of which is increased vascular permeability. The enhanced permeability allows the accumulation of the nanomedicines in ischemic tissue, regardless of the NPs with or without targeting ligands. The released cargo from the nanomedicines further induces the occurrence of neovascularization in ischemic tissue.

A functional vascular system is essential for the proper maintenance of tissues function by delivering oxygen and nutrients, removing waste, and trafficking the transport of immune cells. The interruption of adequate blood causes rapid damage in the ischemic tissue, leading to cell death unless blood supply recovers quickly. Therefore, enhanced neovascularization is essential to establish an equilibrium between energy supply and demand in the ischemic tissues to prevent cell death and tissue dysfunction. Additionally, injured cells in the ischemic tissue can release intracellular chemokines or cytokines which initiate the process of inflammatory response. It is worth noting that the ischemic cascade events occur in a certain sequence and influence one another. According to the reports presented in **Table 1,** nanomedicines that rationally designed for targeting of ischemic tissues by passive and active manner can alleviate the ischemic cascade events, such as oxidative stress, inflammatory, cell death and tissue repair. Typically, the high permeability of blood vessels in ischemic tissue allows the accumulation of nanomedicines to release the payloads, thereby promote vascular regeneration and alleviate inflammatory responses in various ischemic diseases (**Figure 2**). For the dissociation of the cargo from the nanomedicines, we herein do not summarize owing their extensively discussion in many reviews.

#### Passive targeting

When ischemic diseases occur, ischemia leads to vascular dysfunction, one manifestation of which is increased vascular permeability. In recent years, nanoparticles have been used to improve drug delivery to ischemic tissues because of their unique physicochemical properties and their controllable particle size. After intravenous injection, nanoparticles accumulate in damaged tissues through EPR effect. The longer the nanoparticles circulate in the blood, the greater the probability that nanoparticles reach the ischemic area and cross the leaky vessels. Thus, passive diffusion is the main approach for nanoparticles to accumulate in the ischemic lesions. In an ischemic hindlimb model, animal imaging showed that the accumulation of graphene oxide-iron oxide nanoparticles in ischemic tissues reached saturation at day 3 and decreased gradually 91. This result demonstrated that EPR effect resulted in nanoparticle accumulation in the ischemic tissues and passive targeting of nanoparticles could be a potential method for multimodal imaging and therapy of ischemic diseases. Furthermore, the coating and modification of nanoparticles are of tremendous importance for prolonged circulating time, leading to enhanced the bioavailability and ischemic tissue accumulation. Generally, polyethylene glycol (PEG) coating was found to shield the surface of nanoparticles from aggregation and phagocytosis. An example showed that PEGylation of upconversion nanoprobes (UCNPs) effectively protected the particles from immunological recognition, which in turn prolonged the blood circulation time and guaranteed better accumulation efficiency 92.

#### Active targeting

While passive targeting relies on the leakiness of blood vessels, active targeting can be achieved by conjugating ligands on the surface of nanoparticles, enabling site-specific interactions and improving its retention in the target tissues rather than other organs. Of note, the engineered nanomedicines for active targeting cannot avoid passive targeting owing to the enhanced vascular permeability in ischemic tissues. In most ischemic tissue, the disruption of tissue physiology creates an abnormal local environment which can be harnessed for drug delivery. NPs can be modified to respond to specific tissue microenvironment such as low pH and hypoxia 93, 94. A study conducted by Zhang *et al.* designed liposomes by co-assembling indocyanine green for photoacoustic imaging and CREKA (cystein-arginine-glutamic acid-lysine-alanine) peptide for fibrin targeting, which exhibited great capacity to accumulate in the cardiac infarct region 95. In addition, angiotensin II type 1 receptor (AT1R) expression is induced after acute myocardium infarction, which makes it an ideal target for theranostics. For example, angiotensin II-decorated Ag_2_S nanodots was prepared, which showed high affinity for injured endothelial cells overexpressing AT1R 96. In our recent report, we found that the expression of ferritin receptor was enhanced following cardiac ischemia reperfusion (IR). Therefore, we designed a protein-based nanoparticle, ferritin nanocage (FTn), for IR injured tissue targeting 97. Compared to sham heart, the improved accumulation of FTn in IR heart was observed after systemic administration. In addition to direct targeting, indirect targeting also improves the accumulation of nanoparticles in ischemic tissues. During the progression of MI, infiltration and accumulation of macrophages in the ischemic tissues make them as potential targets in cardiac ischemic tissues. Surface modification of iron oxide nanoparticles with dextran allows them targeting macrophages due to the expression of dextran-binding C-type lectins on macrophage surface 98, which provided an indirect method for targeting ischemic tissues.

As a new emerging technology, biomimetic cell membrane coating provides a promising strategy for nanoparticles active targeting to ischemic tissues. Neutrophils and platelets have the ability to target inflammatory areas in response to chemokines 99. For example, neutrophil membrane coating could enhance the accumulation of nanoparticles in the ischemic areas due to the specific interaction of lymphocyte function-associated antigen 1 (LFA-1) on the neutrophil membrane with ICAM-1 that is highly expressed on injured microvascular endothelial cells 100-102. Tang *et al.* reported that neutrophil membrane coated magnetic nanoprobes, composed of PLGA nanoparticles loaded with superparamagnetic iron oxide nanoparticles, exhibited selective targeting to activated endothelial cells in a rodent model of transient middle cerebral artery occlusion 103. Acute MI can also trigger vascular damage and collagen and fibronectin exposure, which specifically recruit platelets with abundant expression of glycoprotein (GP) αIIbβ3 (CD41/CD61) to the injured endothelial cells in ischemic tissues 104, 105. Inspired by this, Li *et al*. fabricated platelet membrane-derived biomimetic nanobubbles for targeting acute ischemic brain tissues, which can be used for early intervention and serve as novel theranostic nanoformulations 106. As a biomimetic top-down strategy, the platelet membrane-assembled nanobubbles resulted in increased targeting ability to stroke lesion, which can furthermore form larger bubbles, enabling detectable ultrasound signals in the lesion area.

### Nanomedicines for ischemic disease therapy

#### Myocardial ischemia

##### Nanomedicines with passive targeting of myocardial ischemia

Accumulating evidence shows that miRNAs may play an essential role in cardiovascular diseases. Previous studies reported that miRNAs were responsible for angiogenesis, such as miR-126, which could block the expression of sprouty-related EVH1 domain-containing protein 1 (SPRED1) that was known to inhibit the angiogenic factor, VEGF 107. Therefore, PLGA nanoparticles encapsulating miR-126 were fabricated, allowing the localized and sustained release of miR-126 for effective control of SPRED1 and promoting angiogenesis. Tissue ischemia is a complex pathological microenvironment and progress over time. Developing an intelligent drug system, which could gradually release drug in response to the ischemic microenvironment, would be therapeutically attractive for treatment of ischemic diseases. In a recent study, Lin *et al*. prepared a biosmart nanoparticle by encapsulating melatonin in the core and plasmids encoding VEGF in shell which was designed to protect cells by releasing melatonin to scavenge reactive oxygen species (ROS) at the acute stage and to secret VEGF for vascularization by sensing hypoxia at the chronic stage 94, 108. Importantly, VEGF secretion with an oxygen-sensing feature, could be up-regulated in hypoxic microenvironment and be shut down in normoxic microenvironment. Such nanoparticles have shown great potential for the treatment of MI and may represent a promising intelligent nanodrug system 108.

##### Nanoparticles with active targeting of myocardial ischemia

**Figure 3:**
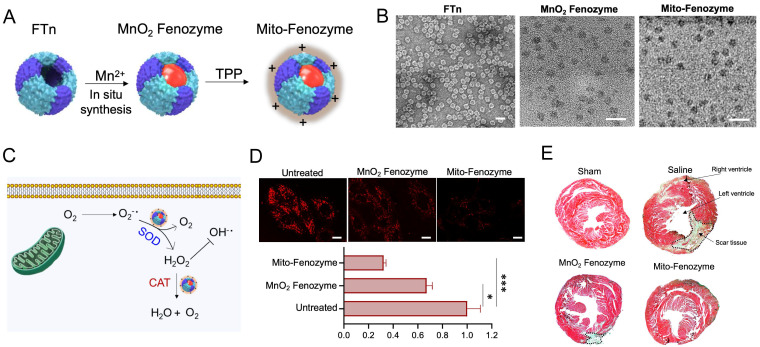
** Mitochondria-targeted hybrid nanozymes as superoxide scavengers protect heart function during IR injury. (A)** Mito-Fenozyme were fabricated by *in situ* synthesis of MnO_2_ into FTn core via Mn^2+^ oxdation in the presence of H_2_O_2_, followed by TPP-NHS ester conjugation with free -NH_2_ of protein. **(B)** TEM images of FTn protein shell (negative staining with 1% uranyl acetate), MnO_2_ and Mito-Fenozyme. **(C)** Schematic of intracellular conversion of free radicals to noncytotoxic molecules under Mito-Fenozyme. **(D)** Confocal images (upper) and quantification analysis (bottom) of the effect of MnO_2_ Fenozyme and Mito-Fenozyme on mitochondrial oxidative injury by mitoSOX as O^2•-^ indicator. Scale bar = 10 µm. **(E)** Representative images of midpapillary regions of the hearts, 14 d after IR using Masson's trichrome staining. Blue, collagen-rich scar tissue; red, viable myocardium. The scar tissue was indicated with dashed box. Adapted with permission from 97, copyright 2021, John Wiley & Sons, Inc.

The mechanisms of IR injury are complicate, but a well-characterized initiating factor is the burst of ROS from mitochondrial respiratory chain upon reperfusion. High concentration of ROS generation from mitochondria resulted in oxidative stress, loss of mitochondrial function and eventually death of cardiomyocytes. Therefore, development of mitochondria-targeting ROS scavenging nanoparticles may be a feasible strategy for the treatment of IR injury. A *de novo* design strategy was proposed that artificial hybrid enzymes were created by *in situ* synthesis of Mn nanoparticles in recombinant human ferritin nanocages (FTn) and modified with a lipophilic cation, TPP, on the FTn surface for mitochondria targeting 97. The hybrid nanozymes showed high enzyme-like activity to scavenge excessive ROS by transformation of excessive mitochondrial O_2_^•-^ into H_2_O_2_ and subsequent conversion of H_2_O_2_ into H_2_O, which endowed protective roles in cardiac IR injury (**Figure 3**).

Exosomes are nano-sized membrane vesicles derived from a variety of cell types and can mediate cell-to-cell communications and convey immune-modulatory and pro-angiogenic signals. Exosomes released by MSCs contain various cargoes including proteins and miRNAs with the potential for angiogenesis and cardiac regeneration. Numerous studies have proved that MSC-derived exosome/extracellular vesicles are versatile delivery systems for the treatment of ischemic diseases. For example, Liu *et al*. found that MSC-derived exosomes significantly inhibited cardiomyocyte apoptosis and reduced the infarct size by inducing cardiomyocyte autophagy via targeting AMPK and Akt pathway 109. It has demonstrated the potential for using exosome/extracellular vesicle as robust and pleiotropic nanocarriers for gene therapy. However, conventional exosomes are prone to nonselective accumulation within the normal organs, escepically lung and liver, leading to insufficiency in targeting myocardial ischemic tissues and possible toxicity to irrelevant organs. Recently, a new petide selectively targeting ischemic myocardium was discovered via phage display technique 110. Furthermore, the fusion with ischemic myocardium-targeting peptide CSTSMLKAC (IMTP) could endow exosomes significant specificity and efficiency to ischemic myocardium. Meanwhile, MSC-derived IMTP-exosomes exert remarkable effect on inhibiting inflammation and enhancing angiogenesis and cardiac functions in a mouse MI model. Thus, ischemic myocardium specific peptide sequences engineered exosomes represent a novel molecular tool which may be useful in targeting and treatment of ischemic myocardium 111. Additionally, a smart nanoparticle was fabricated by a Fe_3_O_4_ core and a silica shell modified with two types of antibodies which could specifically bind CD63 antigen on the exosomes and myosin-light-chain on the injured cardiomyocytes, respectively 112. Under a local magnetic field, accumulation of the nanoparticles and release of captured exosomes under the acidic pH of ischemic cardiac tissues promoted angiogenesis and cardiac repair. However, the small quantities of exosomes secreted from MSCs limited their clinical applications 113, this drawback inspired the development of exosome-like nanovesicles in the application for the treatment of ischemic myocardium. Recently, exosome-mimetic extracellular nanovesicles (NVs) derived from iron oxide nanoparticles-incorporated MSCs (IONP-MSCs) were developed by extrusion that could induce HIF-1α mediated growth factor expression and augment the retention in the infracted heart by magnetic guidance 114. The accumulated IONP-NVs in the infarcted heart significantly inhibited apoptosis and fibrosis, and improved angiogenesis and cardiac function recovery.

**Figure 4:**
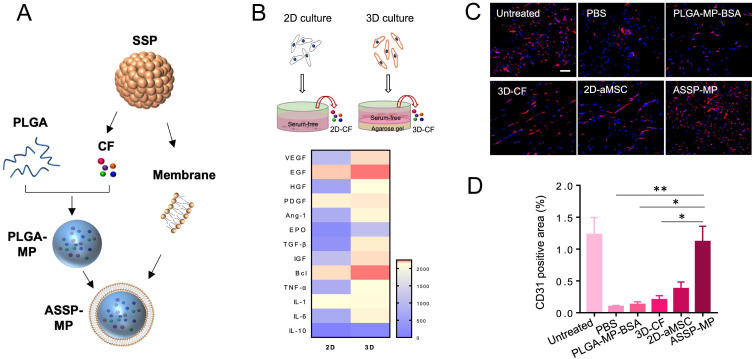
** Cell-like delivery system (ASSP-MP) for improved vascular regeneration in MI model. (A)** ASSP-MPs were fabricated by loading proangiogenic bioactive factors collectively known as“cocktail” factors (CFs) secreted from SSPs into PLGA-MPs, and then PLGA-MPs were coated with cell membrane of SSPs to form ASSP-MPs. **(B)** Heatmap of proteins differentially expressed in 2D-CF and 3D-CF, based on LC-MS proteomic analysis. **(C)** Confocal imaging and **(D)** image-based quantification analysis of blood vessels in ischemic muscles by immunostaining with anti-CD31 antibody at 21 days after treatment. Scale bar = 50 µm. Adapted with permission from 27, copyright 2020, American Association for the Advancement of Science.

Mounting evidences suggest that stem cells exert their therapeutic effects primarily through paracrine mechanism 115. This inspired researchers to build a synthetic stem cell, which mimic the paracrine activity of natural stem cells during tissue repair 116. A novel synthetic cardiosphere-derived cell (CDC) that has a paracrine factor core and a platelet membrane shell with PGE_2_ decoration was designed, which exhibited natural infarct-homing ability in the pathological cardiac microenvironment and sustained release of paracrine factors to promote angiogenesis 117. The construction of the cell mimics increased the stability and bioactivity of paracrine factors. The platelet membrane cloaking approach represented a straightforward and robust method to enhance injury targeting and further augmented the viability and functions of synthetic CDCs. While the therapeutic strategies discussed above have created synthetic stem cells or cell-mimicking composites, there is another groundbreaking innovation based on biomimetic functionality of 3D stem cell spheroid (3D SSPs) derived secretome. Unlike common 2D culture of stem cells, the 3D SSPs mimicking the hypoxic microenvironment showed significantly superior pro-angiogenic capacity. Furthermore, a particle-based artificial stem cell spheroid technology packing paracrine factors of 3D SSPs was fabricated with cell membrane-derived surface coatings for the treatment of ischemic diseases 27. The artificial SSP-NPs coated with RBC/platelet hybrid membrane significantly improved ischemic tissue accumulation, mainly due to the enhanced circulation half-life of RBCs and the specific binding capacity of platelets to injured endothelial cells. The artificial SSP particles provided superior revascularization effects on the ischemic tissues, which offered a promising therapeutic strategy for ischemic tissue regeneration and overcome issues with stem cell therapies (**Figure 4**).

#### Ischemic stroke

##### Nanomedicines with passive targeting of ischemic stroke

Timely blood supply in the cerebral ischemic lesion can reverse tissue damage and inhibit neurological injury. Nanoparticles based angiogenesis gene delivery which could effectively avoid the risk of gene degradation has been developed for successful ischemic stroke therapy. As above mentioned, HIF-1α regulates the transcription of many pro-angiogenic genes. Prolyl hydroxylase 2 (PHD2) silencing alone was demonstrated to stabilize HIF-1α and increase the survival ability of endothelial progenitor cells (EPCs) in ischemic lesions 118. For instance, amphiphilic low molecular weight polyethyleneimine (PEI) encapsulated superparamagnetic iron oxide (SPIO) nanoparticles was successfully prepared for PHD2 siRNA delivery and showed a significantly reduced infarct volume, increased angiogenesis and neurological function 119.

Insufficient secretion of neuroprotective factors and inadequate cells homing to the ischemic cerebrum make MSC-based therapy compromised in practical applications. For the first time, Huang *et al.* found that iron oxide nanoparticles themselves upregulated CXCR4 expression of MSCs by mediating HIF-1α expression, which improved MSC homing to injured area 120. Based on this finding, a recent study presented a simple and effective approach to genetically engineer MSCs using one dimensional assembled magnetosome-like ferrimagnetic iron oxide nanochains (MFIONs) to upregulate CXCR4 for promoting MSCs homing to the ischemic cerebrum and to overexpress brain-derived neurotrophic factors for ischemic cerebrum function recovery 121. This effective and safe strategy demonstrated great potential for the current treatment of post-stroke recovery.

For patients who have microcirculation impairment, O_2_ delivery to the ischemic cerebral tissues is of pivotal importance 122. The regulation of oxygen content is crucial after ischemia since either hypoxia before thrombolysis or an explosive rise of O_2_ after thrombolysis lead to serious impairment. Hemoglobin (Hb) that is a tetramer with four oxygen-binding sites has showed great potential as an oxygen reservoir in the application of regenerative medicine for oxygen supply. However, the short half-life limits the clinical application of Hb. Liposomal Hb could not only prolong the circulation time of Hb *in vivo*, but also deliver sufficient O_2_ to microcirculation where red cells seldom reach 108. Further loading an engineered Mn_3_O_4_ nanosponge that could release oxygen and scavenge ROS was proposed. This strategy represents an attractive design to regulate the microenvironment of ischemic stroke.

##### Nanomedicines with active targeting of ischemic stroke

**Figure 5:**
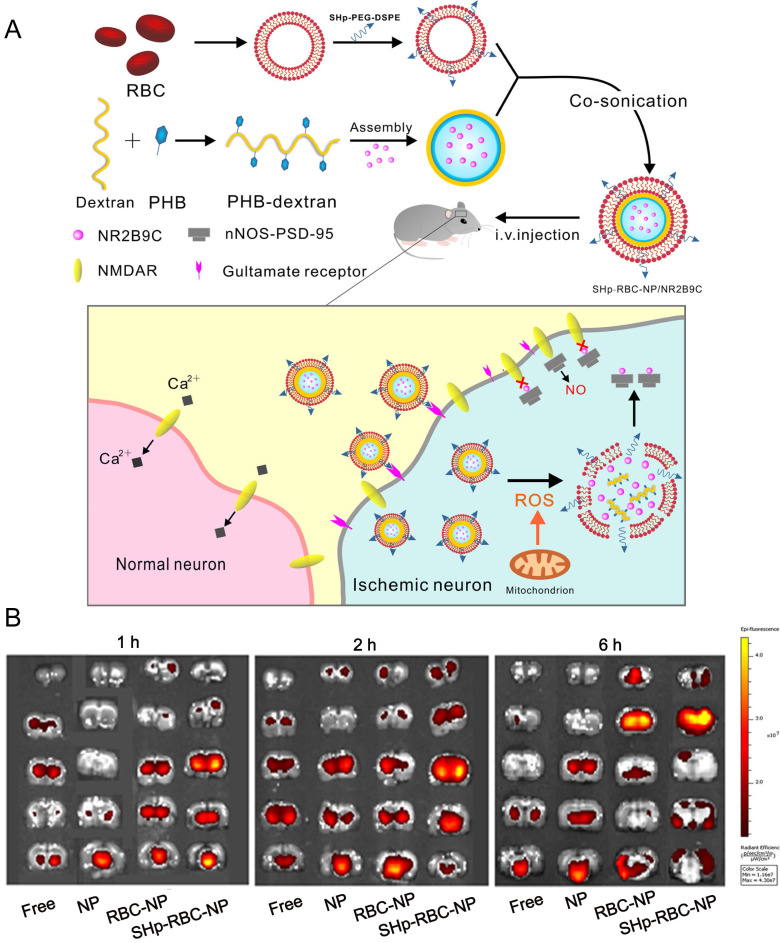
** Bioengineered Boronic Ester Modified Dextran Polymer Nanoparticles as Reactive Oxygen Species Responsive Nanocarrier for Ischemic Stroke Treatment. (A)** Schematic design of the SHp-RBC-NP/NR2B9C. After intravenous injection, the SHp-RBC-NP/NR2B9C could prolong the circulation life with the RBC-mimicking properties and then target to the ischemic brain site via stroke homing peptide mediated transcytosis. **(B)** *Ex vivo* fluorescent image of rhodamine-labeled free NR2B9C, NP, RBC-NP, and SHp-RBC-NP in the ischemic brain sections at 1, 2, and 6 h, respectively. Adapted with permission from 130, copyright 2018, American Chemical Society.

Currently, drug delivery nanoparticles for the treatment of ischemic stroke are less than satisfactory, mainly due to the presence of BBB and the short circulation time of nanoparticles. It has been reported that ischemia contributes to BBB opening, which is attributed to endothelium activation, leukocyte recruitment, cytokine production and edema formation 123. Although previous studies showed that the permeability of the BBB was increased when the occurrence of ischemic stroke, it remained as obstacles for drugs delivery. Importantly, it remains unclear whether the enhanced permeability is size-dependent. Besides the increased leakage of BBB, ischemia can induce the expression of some specific receptors on endothelial cells which also raised the opportunity to increase the rates of NPs across the BBB. Active targeting strategies involve conjugating ligands to NP surface to facilitate uptake via specific BBB receptors, which could enhance the effects of passive extravasation. Until now, several targets have been reported for NP-mediated drug delivery bypassing the BBB, such as transferrin receptor (TfR), low density lipoprotein receptor-related proteins (LRP) 124, insulin receptor (IR) 124 and lipoprotein receptors (low density lipoprotein receptor (LDLR) 125. Additionally, ligand density is of great importance in determining BBB transport efficacy. In general, surface functionalization of NPs with an intermediate number of weakly binding ligands may be more likely to achieve optimal transport across the BBB. It has been reported that AuNPs functionalized with low concentrations of Tf (20-30 molecules per NP) was more effective for BBB penetration than conjugating with high concentrations of Tf (100-200 molecules per NP). Further studies revealed that AuNPs entered brain primarily in a TfR-mediated process, when AuNPs with a low ligand density were able to interact effectively with the TfR and accumulate in the parenchyma to a significant extent, whereas those with a high ligand density failed to induce effective transcytosis 126. Collectively, these results suggest that regulating ligand targeting has the potential to enhance the efficacy of NP-mediated brain penetration. For example, chitosan NPs conjugated with TfR antibody and loaded with a specific caspase-3 inhibitor (ZDEVD-FMK), showed promising results. This formulation was able to cross the BBB, and decreased infarct volume and neurological deficits induced by ischemia stroke 127. Integrins involving in the maturation of vessel lumen are found widely present on the active cerebral endothelial cells. Inspired by this, an active targeting nanoparticle system was constructed by surface conjugation of a PHSRN peptide (Pro-His-Ser-Arg-Asn) which is an integrin α_ν_β_1_ specific binding ligand. In addition, smoothened agonist, an activator of sonic hedgehog signaling, was released in response to the acidic environment for angiogenesis promotion and neurological function recovery 128.In another example, Deng *et al*. conjugated RGD peptides (arginine-glycine-aspartic acid) with hyperbranched cationic polymer (DMAPA-Amyp) to prepare RGD-DMAPA-Amyp nanoparticles for HIF-1α plasmid delivery, which could target injured endothelial cells and accumulate in the ischemic cerebral tissues 129. This delivery system exhibited excellent angiogenesis capacity and improved the recovery of neurological function in zebrafish and rat models. Recently, a smart bioengineered drug delivery nanocarrier was developed for stroke-specific delivery of a ROS scavenging agent, NR2B9C, by coating the RBC membrane modified with the stroke homing peptide (SHp, CLEVSRKNC) 130. For active targeting strategies for the treatment of ischemic stroke, SHp was identified and optimized by phage display in a focal cerebral ischemia rat model. SHp preferentially targeted to ischemic brain tissue with negligible distribution into non-ischemic tissues. Mechanismly, SHp can specifically target to a portion of neuronal cells undergoing apoptosis at the penumbra region of ischemic brain tissue. Therefore, SHp modified nanoparticles could improve drug accumulation in ischemic brain area. This system (named as SHp-RBC-NP) may represent a promising intervention for the treatment of ischemic stroke in the clinic (**Figure 5**).

The administration of MSCs has attracted great attention for the treatment of ischemic stroke, whereas the low delivery efficiency of MSCs to the stroke lesion limits their clinical application 122, 131, 132. Furthermore, even though cells accumulated in the ischemic tissues after systemic injection, they can hardly survive in hypoxic and inflammatory conditions. Exosomes showed significant therapeutic efficacy in the treatment of ischemic stroke and their nano-size helps their accumulation in the ischemic lesion of brain 133, whereas the small quantities of exosomes secreted from MSCs limited their clinical application. A previous study demonstrated exosome-mimetic NVs prepared by extruding IONP-harbored MSCs through serial nano-porous membrane filters exhibited increased production yield compared to naturally exosomes. The magnetic nanovesicles (MNVs) were further administrated to the ischemic lesion with guidance of magnetic navigation in a rat ischemic stroke model. The MNVs showed to promote angiogenesis, anti-apoptosis and anti-inflammatory response and reduced neuronal damage of the ischemic stroke 131.

Endothelium-derived nitric oxide (NO), as a vessel-functional molecule, can regulate vasodilation, inhibit the aggregation of platelet and leukocytes and promote angiogenesis. Therefore, targeted delivery of NO to the ischemic lesion can recanalize the blocked microvascular. Based on this, a biomimetic nanoparticle was constructed by a platelet membrane encapsulated with L-arginine and γ-Fe_2_O_3_ magnetic nanoparticles (PAMNs). The PAMNs specifically targeted and accumulated at the ischemic tissues under an external static magnetic field gradient and produced NO *in situ* after tail vein administration, which promoted the revascularization and blood flow 134. Neutrophils have unique properties which enable targeting inflammation sites by interacting with injured microvascular endothelial cell. In one example, mesoporous Prussian blue nanozymes coated with neutrophil cell-membrane (MPBzyme@NCM) were reported for the treatment of ischemic stroke. MPBzyme@NCM could promote microglia polarization toward M2, enhance delivery of the nanozymes to injured area and relieve ischemic damage 135. This biomimetic strategy improved the delivery efficiency of nanoparticles to the damaged brain and provided an attractive perspective for ischemic diseases.

#### Hindlimb ischemia

##### Nanomedicines with passive targeting of hindlimb ischemia

Previous study has demonstrated that conjugation of VEGF on gold NPs maintained VEGF bioactivity, promoted new blood vessels formation and increased blood perfusion which reached over 90% of normal tissues, whereas the same amount of free VEGF did not result in any significant improvements. In another instance, using the supramolecular self-assembly strategy, a peptide from insulin-like growth factor-1(IGF-1) folded into 3D supramolecular nanomaterials which exhibited a low dissociation constant to IGF-1 receptor and efficiently activated the IGF-1 downstream pathway. More importantly, the formation of supramolecular nanofibers showed superior bioactivity over native IGF-1 due to the enhanced biological stability 136. Protein delivered by nanoparticles degraded gradually, whereas delivery of plasmids encoding pro-angiogenic factors can sustainably produce angiogenic factors without multiple injection which has shown encouraging preclinical outcomes. In a rabbit ischemic hindlimb model, magnetic gelatin nanospheres encapsulated with VEGF plasmids were intra-arterially injected and magnetically targeted to the ischemic site, leading to 50% increase of blood vessel density compared to nanospheres without VEGF plasmids 137.

The establishment of angiogenesis typically takes days to weeks, which is largely unable to meet the requirement of revascularization of ischemic tissues, especially in the circumstance of acute ischemic events. Thus, there is an urgent need for developing oxygen-release system to elevate tissue oxygen level and improve cell survival. Recently, researchers developed an oxygen-release system by combination of hydrogel with microspheres based on a core-shell structure with PVP/H_2_O_2_ as core and PLGA as shell. Oxygen release was dependent on the diffusion of PVP/H_2_O_2_ complex through PLGA shell, and then H_2_O_2_ was turned into O_2_ by catalase embedded in gelation hydrogel. The duration of oxygen releasing lasted 4 weeks, which augmented survival of injured cardiac cells 138. While the system was potential, excessive O_2_ may lead to excessive ROS formation since O_2_ cannot been released controllably in response to tissue oxygen concentration. Furthermore, Guan *et al*. developed microspheres with an oxygen responsive shell which contained 2-nitroimidazole whose degradation rate increased when the environmental oxygen level decreased. This property allowed the microspheres to release O_2_ when sensing hypoxic microenvironment. When co-delivery of the microspheres and MSCs to the mouse ischemic hindlimb, this system was capable of releasing O_2_ controllably and increased MSCs survival and their paracrine effects 139. This strategy represented a smart method of delivering O_2_ to ischemic tissues to improve endogenous and exogenous cell survival, which can avoid cell death causing by excessive oxygen release.

##### Nanomedicines with active targeting of hindlimb ischemia

Recent studies showed that MSCs homing to ischemic tissues is mediated by interaction between stromal-derived factor (SDF) secreted by injured tissues and CXCR4 expressed on MSCs 140. With the goal of improving targeted delivery of nanocarriers to ischemic hindlimb, researchers functionalized nanocarriers with engineered MSC membrane overexpressing CXCR4, leading to improved tropism of nanocarriers towards the ischemic tissues. When further loaded with VEGF, the nanocarriers modified with genetically engineered membrane exhibited substantially enhancement of blood reperfusion, tissue repair, and limb salvage compared to animals treated with nanocarriers loaded with similar concentration of VEGF without engineered stem cell membrane coating.

Unlike *in vitro* conventional culture, the secretome and membrane composition of MSCs which suffer inflammatory and hypoxic conditions* in vivo* in the infarct area were absolutely different. We recently constructed 3D MSCs spheroids to mimic the hypoxia microenvironment, and the differences of protein content were compared between 3D MSCs spheroids and 2D MSCs. Vascular regeneration-associated proteins were the topmost elevated proteins identified in 3D cell spheroids. Furthermore, a microparticle system by integration the secreted factors from 3D cell spheroids with cell membrane-derived coating was developed and showed superior revascularization effects in hindlimb ischemia model 27. Most recently, instead of loading growth factors to promote angiogenesis, we constructed a novel delivery system with surface coating of copper-containing protein to timely release nitric oxide (NO) 141. A rational designed nanovesicle was achieved by collection both of membrane and secretome from MSCs cultured in the presence of inflammatory cytokines via a one-step extrusion strategy 142. It has been demonstrated that the nanocarriers, so called Meseomes, could mimic MSCs functionality in tissue repair and immunoregulation, showing improvement of angiogenesis, reduction of fibrosis and inflammation in the ischemic hindlimb model. Meseomes represented a smart biomimetic and therapeutic agent which possessed the tailored biological functions meeting the demands of the ischemic diseases 142.

## Conclusion and outlook

Therapeutic vascular regeneration is essential for ischemic diseases but remains a major challenge. Direct delivery of the factors (*e.g.*, VEGF) tends to disequilibrate physiological states, leading to various proinflammatory diseases. In addition, the complex angiogenic factor cascades are currently mapped only incompletely. There are many efforts to develop pro-angiogenic drug-based therapies in recent years, but only a single pro-angiogenic drug has reached the market. Regranex that contains recombinant human PDGF-BB was approved for the treatment of diabetic foot ulcers 143. However, owing to its limited efficacy, Regranex was used at supraphysiological doses, which increased the cost and raised important safety concerns, especially for an increased risk of systemic cancer. Thus, strategies to deliver growth factors are difficult to mimic the spatio-temporal complexity of an angiogenic response. Instead, utilization of hypoxia-induced energy pool as an angiogenic tool has considered to be a leading strategy by naturally mimicking the angiogenesis process in physiological (*e.g.*, embryogenesis), as well as pathological (*e.g.*, ischemia, wound healing, tumor formation) states. In other words, modulation of hypoxic status in disease sites or delivery of hypoxia-mastering cocktail factors would be an ideal choice for inducing vascular regeneration in ischemic tissues.

Until now, the nanomedicines have not achieved clinical translation for treatment of ischemic diseases. In fact, the development of nanomedicines for ischemic diseases remains in its infancy, and there are many issues need to be addressed. i) Detailed mechanism of enhanced vascular permeability of ischemic tissues. Several aspects are still unclear, such as size cutoff of nanoparticles and dynamic permeability changes after ischemia, which guide to design the desired nanomedicines and understand optimal therapeutic time window. ii) Choice of surface markers for active targeting. Currently, the available surface markers in ischemic tissues are limited, leading to the lack of design principles for active targeting of nanomedicines. Nanoparticles intravenously administered into body can be delivered to the disease regions via passive targeting and/or active targeting. Passive circulation and extravasation of NPs are dependent on their blood circulation time and the accumulation in specific disease regions as a result of certain pathological conditions. Although this rationale for the design of passive targeting NPs is well accepted, recent studies suggest that passive targeting is not always effective and reliable for nanoparticle delivery. It may be compromised by a number of biological barriers, such as kidney and lung epithelia, blood-brain barrier. Whereas, functionalized NPs with targeting ligands can enhance the effects of passive diffusion due to improving disease regions specificity while minimizing unwanted off target effects 144. Furthermore, nanomedicines with targeting ability could not only increase the therapeutic effect, but also reduce the injection dose and toxicity of drugs. iii) Spatio-temporal modulation strategy. The occurrence and progression of ischemic diseases involve multiple pathological mechanisms, including vascular occlusion, oxidative stress, and inflammatory response 145. Due to the spatial and temporal distribution patterns of endogenously produced pro-angiogenic factors, it is important to create more complex nanosystems with the ability to modulate the pro-angiogenesis effect in a spatial and temporal manner. iv) Administration dose and frequency. Ischemic cascade events occur in a certain sequence and influence one another. A great number of nanomedicines have been designed with specific functions against pathological events at different stages of the ischemic diseases 146. Therefore, intervening the different periods of ischemic cascade required different administration dosage and frequency of nanomedicines. Moreover, the properties of nanocarrier, including surface charge, particle size, shape and hydrophilic characteristics would affect the fate of nanomedicines *in vivo*. Currently, there is no uniform standard dose and frequency of the administration of nanomedicines as illustrated in **Table 1**, requiring extensive investigations. Indeed, nanomedicine is a promising field for the treatment of ischemic diseases, but more investigations are needed to eventually make the transition to clinical practice. Although there are no clinical investigations on nanoparticle based-therapeutics currently, nanomedicines are still a promsing technology to combine with other therapies for synergistic treatment of ischemic diseases.

## Figures and Tables

**Table 1:** Nanomedicines designed for passive and active targeting of ischemic tissues

NP type	Size	Surface modification	Disease	Delivery of payload	Targeting manner	Dose	Time of administration	Therapeutic mechanism	Reference
PLGA	205 nm	PGE_2_ modified platelet membrane	myocardial ischemia/reperfusion	CDCs paracrine factors	active targeting	CDCs secretome dose: 1.2 mg kg^-1^, PGE_2_ dose: 0.053 mg kg^-1^	every 7 days for 4 weeks	pro-angiogenesis	117
Fe_3_O_4_ core and silica shell	20 nm	anti-CD63 and anti-MLC antibodies	myocardial ischemia	exosomes	active targeting	Rat: 10 mg kg^-1^ Rabbit: 2.5 mg kg^-1^	2 weeks after ligation	pro-angiogenesis	112
PLGA	125 nm	Red cell membrane and platelet membrane	myocardial ischemia/hindlimb ischemia	SSP-secreted factors	active targeting	3D-CF dose: 1.2 mg kg^-1^	24 hours after surgery, additional 3 doses injected every 1 week.	pro-angiogenesis	27
Mitochondria-inspired nanoparticles	170-190 nm		myocardial ischemia	VEGF and melatonin	intramyocardial injection	2.5 mg of NPs	immediately after ligation	pro-angiogenesis and scavenging ROS	94
Liposomes	142 nm	AT1 ligand	Myocardial ischemia		active targeting	100 μl nanoparticles (∼1.5 × 10^14^ particles/mL)	1, 4, 7 days post-MI	Fluorescence imaging	147
PLGA	74.4 nm	polyethylenimine (PEI)	myocardial ischemia	IGF-1	intramyocardial injection	IGF-1 dose: 20 ng in 20 μl PBS	immediately after coronary artery ligation	prevent cardiomyocyte apoptosis	148
PLGA	5.01 ± 0.23 μm	PEG	myocardial ischemia	NRG1 and FGF1	intramyocardial injection	growth factor dose: around 1675 ng	at 4 days post MI induction	pro-angiogenesis	149
Ag_2_S nanodots	9 nm	angiotensin II peptide (ATII)	myocardial ischemia/reperfusion		active targeting	Ag_2_S nanodots dose: 0.3 mg	after reperfuion and then circulation for 30 min	Infrared fluorescence imaging	96
FTn	12 nm	TPP	myocardial ischemia/reperfusion	MnO_2_	active targeting	Mito-Fenozyme dose: 2.5 mg kg^-1^	3 doses every 2 days	scavenging ROS	97
IONP-NVs	70-140 nm	MSC membrane	myocardial ischemia	MSC-derived nanovesicles with IONPs	intramyocardial injection	IONP-NVs dose: 150 μg	1 day after the ligation	pro-angiogenesis	114
high-loading peptide nanoparticles (HLPNs)	6.6 ± 0.8 nm		myocardial ischemia-reperfusion	ExE motif of integrin β3 derived peptide (M3mP6)	active targeting	M3mP6 HLPNs dose: 5 or 10 μmol kg^-1^	35 minutes after occlusion	preventing thrombosis	150
platelet membrane-derived biomimetic nanobubbles (PNBs)	131.43 ± 19.84 nm	platelet membrane	ischemic stroke	SF_6_ gas	active targeting	PNBs dose: 200 μl	immediately after the ligation	ultrasound imaging	106
neutrophil membrane-coated nanoprobes (NMNPs)	159.9 nm	neutrophil membrane	ischemic stroke	SPIO	active targeting	NMNPs dose: 20 nmol kg^-1^	at 24 h post-reperfusion of the MCAO model	magnetic resonance imaging	151
MNVs	194.2 ± 44.5 nm	MSC membrane	ischemic stroke	MSC-derived nanovesicles with IONPs	active targeting	MNVs dose: 200 μg		pro-angiogenesis and anti-inflammation	131
PAMNs	200 nm	Platelet Membrane	ischemic stroke	L-arginine and γ-Fe_2_O_3_	active targeting	PAMNs dose: 200 μl (1.41 ± 0.16) × 10^8^/mL MNs dose: 71.46 μg mL^-1^.		pro-angiogenesis	134
SAG@PHSRN-HES	31.52 nm	Pro-His-Ser-Arg-Asn (PHSRN) peptides	ischemic stroke	smoothened agonist (SAG)	active targeting	SAG dose: 20 mg kg^-1^	1 day after infarction, for 5 consecutive days	pro-angiogenesis and neurological function recovery	128
“nanoplatelet”	167.2 ± 1.6 nm	platelet membrane	ischemic stroke	recombinant tissue plasminogen activator (rtPA) and neuroprotectant (ZL006e)	active targeting	ZL006e dose: 4 mg kg^-1^ and rtPA dose: 0.5 mg kg^-1^	immediately after the ligation	thrombolysis and neuroprotection	152
PEG-UCNPs	45 nm	PEG	ischemic stroke	Gd	passive targeting	Gd dose: 5 mg kg^-1^	after the ligation	magnetic resonance imaging.	92
Alkyl-SPIO/siPHD2	80-120 nm		ischemic stroke	siPHD2	left ventricular injection	5 × 10^5^ EPCs with siPHD2-EPCs	1 day after the stroke induction	magnetic resonance imaging	119
Mn_3_O_4_@nanoerythrocyte-T7 (MNET)	216 nm	T7 peptide	ischemic stroke	Mn_3_O_4_	active targeting	MNET dose: 2 ml of 2.98 × 10^13^ particles/L	after 0.5 h post-MCAO surgery	scavenging ROS and timely oxygen supply	108
RGD-DMAPA-Amyp nanocarrier	100-350 nm	RGD	ischemic stroke	HIF-1α-AA plasmid DNA	active targeting	600 μg/mL,300μl	30 min after cerebral infarction	pro-angiogenesis	129
SHp-RBC-NP/NR2B9C	163.3 ± 4.6 nm	stroke homing peptide (SHp)	ischemic stroke	neuroprotective agent (NR2B9C)	active targeting	SHp-RBC NP/NR2B9C dose: 0.3 mg kg^-1^	daily for 7 days	neuroprotection	130
ultrasmall particles of dextran-coated iron oxide (USPIOs)	20-40 nm	dextran	ischemic stroke	IONPs	active targeting	200 mmol Fe/ml solution, 500 μl USPIOs	5 h after MCAO	magnetic resonance imaging	98
MPBzyme@NCM	160.7 ± 6.08 nm	Neutrophil-like Cell-Membrane	ischemic stroke	Prussian blue nanozyme	active targeting	MPBzyme@NCM dose: 20 mg kg^-1^	1 h after suture extraction	scavenging ROS and neuroprotection	135
polymeric nanocarrier (EPC-CM-NP)	120 ± 20 nm	PEG	hindlimb ischemia	cell conditioned medium of EPCs	intramuscular injections	500 μl EPC-CM-NP containing PDGF-BB dose: 9.115 pg; VEGF dose: 16.47 pg IL8 dose: 35.435 pg	immediately after ischemia induction, additional 2 doses after 1 week and 2 weeks	pro-angiogenesis	153
Cerium oxide nanoparticles (CNPs)	19.5 nm		hindlimb ischemia	Ce^3+^ and Ce^4+^	intramuscular injections	CNPs dose: 0.15, 0.3, and 0.6 mg in 300 μl PBS	on the day after arterial dissection	scavenging ROS, Hif-1α activation and pro-angiogenesis	154
Meseomes	200 nm	MSC membrane	hindlimb ischemia	MSCs secretomes	*in situ* injections	Meseomes dose: 50 μg	every other day for a total of 3 injections	pro-angiogenesis and anti-inflammation	142
ICG-BM nanoparticles	500 nm	Cys-Arg-Glu-Lys-Ala (CREKA) peptides	hindlimb ischemia	indocyanine green-loaded boronated maltodextrin	intramuscular injection	ICG-BM nanoparticles dose: 5 mg/kg	after the induction of ischemia	Ultrasound and photoacoustic imaging; anti-inflammatory and pro-angiogenesis	155
Magnetic DNA nanospheres	13.26 ± 2.72 nm		hindlimb ischemia	VEGF plasmids	active targeting	2 ml DNA-gelatin nanospheres containing 200 μg of plasmids	10 days after ischemia	Pro-angiogenesis	137
Oxygen-release microspheres (Gel/MSC/ORM)	5 μm		hindlimb ischemia	PVP/H_2_O_2_	intramuscular injection	Gel/MSC/ORM dose: 2.5 mg	30 minutes after ligation	Pro-angiogenesis	139
